# Prevalence of Anxiety and Depression Symptoms Among Patients With Inflammatory Bowel Disease: A Multicenter Study

**DOI:** 10.1002/jgh3.70270

**Published:** 2025-09-07

**Authors:** Mohammed Momin Sugie, Gebeyehu Tessema Azibte, Ahmed Adem, Asnake Limenhe, Biruk Abate Legesse, Zekarias Seifu Ayalew

**Affiliations:** ^1^ Department of Internal Medicine Dire Dawa University College of Medicine Dire Dawa Ethiopia; ^2^ Department of Internal Medicine Addis Ababa University College of Medicine Addis Ababa Ethiopia; ^3^ Division of Gastroenterology and Hepatology, Department of Internal Medicine Addis Ababa University College of Medicine Addis Ababa Ethiopia; ^4^ Department of Psychiatry Addis Ababa University College of Medicine Addis Ababa Ethiopia

**Keywords:** anxiety, disease activity, inflammatory bowel disease, mental health

## Abstract

**Background:**

Global evidence has observed that individuals with inflammatory bowel disease (IBD) are at a heightened risk of experiencing psychiatric disorders, which often coincide with a decrease in their quality of life.

**Objective:**

To assess the prevalence of anxiety and depression symptoms and associated factors among patients with IBD.

**Methods:**

An institutional‐based cross‐sectional study was conducted at Tikur Anbesa Specialized Hospital and Adera Medical Center. Categorical variables were presented using frequency and percentage and compared between groups using the chi‐square test. The normal distribution of the continuous variables was assessed using the Shapiro–Wilk test. Mean and standard deviation were calculated for normally distributed data, while the median and interquartile range were calculated for skewed data. We used univariate and multivariate binary logistic regression analysis to examine the factors associated with depression and anxiety; results were reported as adjusted odds ratios (OR) with 95% confidence intervals and *p* value < 0.05 considered statistically significant.

**Results:**

The prevalence of anxiety and depression was 5.1% and 7.1%, respectively, in IBD. Moderate disease activity (AOR = 16.1 (1.7, 156.7), *p* = 0.015) and severe disease activity (AOR = 49.8 (2.1, 1144.02), *p* = 0.014) had a statistically significant association with increased rates of depressive symptoms. Moderate disease activity (AOR = 9.9, 95% CI: 0.9, 106.2, *p* = 0.058) had a positive association. Severe disease activity (AOR = 45.3, 95% CI: 2.0, 1018.0, *p* = 0.016) has a significant associated factor with having anxiety symptoms.

**Conclusion:**

The prevalence of anxiety and depressive symptoms in this study was generally low but can increase due to important factors such as disease activity, smoking, unemployment, a short duration after diagnosis, and having ulcerative IBD.

## 1 Introduction

Inflammatory bowel disease (IBD) is a chronic medical illness that has contributed to a significant burden on global mortality, morbidity, and disability worldwide [1]. The pathogenesis of IBD, which includes ulcerative colitis (UC) and Crohn's disease (CD), is complex and multifactorial. Genetic susceptibility, environmental triggers, alterations in the gut microbiome, and dysregulated immune responses all play a role in the development and progression of these chronic inflammatory conditions [2, 3]. The primary treatment approaches for IBD consist of immunomodulators, biologics, and monoclonal antibodies like anti‐integrins and anti‐interleukins. Starting treatment early can prevent relapses and complications, reduce the risk of malignancy and infections, enhance quality of life, and lessen the occurrence of psychiatric disorders associated with IBD [4].

IBD profoundly affects individuals' quality of life through increased rates of psychiatric disorders [5, 6, 7], medical‐related complaints, and medical costs. Research from around the world indicates that individuals with IBD experience notably higher rates of depression and anxiety compared to the general population [6, 7, 8, 9]. These psychiatric disorders linked to IBD have been shown to diminish quality of life, complicate the treatment of the disease, reduce medication adherence, and heighten the occurrence of active disease [9]. Although traditional belief suggested that IBD primarily affected Western populations, recent evidence indicates a projected exponential rise in the number of IBD cases in low‐ and middle‐income countries, potentially reaching levels seen in the Western world by 2025 as a result of population growth [10].

Evidence from a meta‐analysis of 171 studies with a total of 158,371 participants was found that approximately 35% of IBD patients experienced symptoms of anxiety, while 22% reported symptoms of depression [6, 9]. Another extensive global evidence, in a meta‐analysis of 58 observational studies, was discovered that the pooled prevalence of anxiety symptoms was roughly 32%, and in a meta‐analysis of 75 observational studies, the combined prevalence of depression symptoms was 25% [8, 9]. These psychiatric disorders linked to IBD have been shown to diminish the quality of life, complicate the treatment of the disease, reduce medication adherence, and heighten the occurrence of active disease [9].

Screening and monitoring psychological disorders in patients with IBD play a significant role in both primary care and specialist settings. Unfortunately, no IBD‐specific instruments to measure anxiety or depression disorders have been validated to date. One approach to determining anxiety and depression is to use existing screening measures that have established reliability and effectiveness, which are easy to score and can be coded into electronic medical records. One of these tools is the Generalized Anxiety Disorder Scale (GAD‐7) [11] and Patient Health Questionnaire (PHQ‐9) [12]; multipurpose, self‐reported screening tools; validated and found reliable for use in screening, monitoring, and measuring the severity of anxiety and depressive symptoms.

This study highlights the significant impact of IBD on mental health. It emphasizes the importance of healthcare providers understanding this connection to deliver better care, including timely interventions and personalized treatment plans. The study also calls for policymakers to recognize the burden of depression and anxiety among IBD patients and allocate resources accordingly. By understanding these psychiatric symptoms, policymakers can develop strategies to improve access to care, enhance healthcare infrastructure, and fund research initiatives. Additionally, the study provides evidence for health equity advocates to advocate for addressing disparities in healthcare access and outcomes. Overall, the findings emphasize the need for a broader perspective on IBD management that addresses both physical and mental health aspects.

## 2 Methodology and Materials

### 2.1 Study Design

A cross‐sectional analytical hospital‐based study was conducted at Tikur Anbessa Specialized Hospital and Adera Medical Center from June 2023 to September 2023.

### 2.2 Inclusion and Exclusion Criteria

#### 2.2.1 Inclusion Criteria

- IBD patients, including Crohn's disease and/or Ulcerative colitis, whose diagnosis is well established and have follow‐ups at Tikur Anbessa Specialized Hospital and Adera Medical Center
- IBD patients who are above the age of 18

#### 2.2.2 Exclusion Criteria

- Co‐morbid chronic medical illnesses like diabetes, heart failure, chronic kidney disease (CKD), asthma, and/or chronic obstructive pulmonary disease (COPD), malignancy, and retroviral infection (RVI) prior known psychiatric illnesses.

### 2.3 Sample Size and Sampling Technique

The sample size is calculated using 50% as the prevalence of anxiety and depression since we do not have a prior study done in the same area. A 95% level of certainty and a maximum discrepancy of 5% were considered.

$$N={\left(\mathrm{Z\alpha}/2\right)}^{2}\times p\left(1-p\right)/d^{2}$$

where *N* is sample size; *Z* is standard proportion population at 95% confidence interval (1.96); *P* is estimated proportion of anxiety and depression (50%); *d* = margin of error (5%); sample size, *N* = 384. However, since our sample population is less than 10,000, our required minimum sample will be 384/(1 + (384/150)) = 108. Then we will add a 10% non‐response rate to get the final sample size = 118.

### 2.4 Data Collection Procedure and Methods

Data were collected using a standardized questionnaire used to screen for Anxiety and Depression symptoms. They were adapted from previously published studies on the same topic and are valid in our locality. One of these tools is the Generalized Anxiety Disorder Scale (GAD‐7), an anxiety screening tool that has sensitivity and specificity of 89% and 82%, respectively, to pick generalized anxiety disorder. The Patient Health Questionnaire (PHQ‐9) is another multipurpose, self‐reported screening tool validated and found reliable for screening, monitoring, and measuring the severity of depressive symptoms. It has shown sensitivity and specificity of 88% for major depression. Trained data collectors collected data under the supervision of the Investigator. Data collectors were selected from among the hospitals' GI unit nursing staff in both centers. Data collectors received training from the principal investigators on obtaining the required information from patients attending the GI clinic at both centers.

### 2.5 Operational Definitions

Patients were labeled as having significant anxiety symptoms based on the GAD‐7 screening tool for anxiety (score ≥ 10).

Patients were labeled as having significant depressive symptoms based on the PHQ‐9 screening tool for depression (score ≥ 10).

Those patients on follow‐up who met standard criteria for CD and/or UC based on a combination of investigations (including clinical, biochemical, stool, endoscopic, imaging, and histopathology reports) were labeled as having IBD.

### 2.6 Crohn Disease Activity Index (CDAI) Interpretation

0–149 points: Asymptomatic remission.

150–220 points: Mildly to moderately active Crohn disease.

221–450 points: Moderately to severely active Crohn disease.

451–1100 points: Severely active to fulminant disease.

### 2.7 Ulcerative Colitis Disease Activity Interpretation

#### 2.7.1 Mild

Patients with mild clinical disease have ≤ 4 stools per day with or without small amounts of blood, no signs of systemic toxicity (e.g., no tachycardia), and a normal C‐reactive protein (CRP) and/or erythrocyte sedimentation rate (ESR). Mild crampy abdominal pain, tenesmus, and periods of constipation are also common, but severe abdominal pain, profuse bleeding, fever, and weight loss are not part of the spectrum of mild disease.

#### 2.7.2 Moderate

Patients with moderate clinical disease may have frequent (four to six per day), loose, bloody stools, mild anemia not requiring blood transfusions (hemoglobin > 10 g/dL), and abdominal pain that is not severe. Patients have no or minimal signs of systemic toxicity. Adequate nutrition is usually maintained, and weight loss is not associated with moderate clinical disease.

#### 2.7.3 Severe

Patients with a severe clinical disease typically have frequent, loose, bloody stools (≥ 6 per day) with severe cramps and evidence of systemic toxicity as demonstrated by fever (temperature ≥ 37.8°C), tachycardia (heart rate ≥ 90 beats per minute), anemia (hemoglobin < 10 g/dL), and/or an elevated CRP or ESR. Patients may have weight loss.

### 2.8 Statistical Analysis

The categorical variables in the study were presented using frequency percentages and compared between groups using the chi‐square test. The normal distribution of continuous variables was assessed using the Shapiro–Wilk test. The mean and standard deviation were calculated for normally distributed data, while the median and interquartile range were calculated for data with a skewed distribution. A multicollinearity test was performed for categorical, continuous, and binary variables. Multicollinearity is measured by variance inflation factor (VIF) and tolerance. When a VIF was below five, and tolerance was above 0.1, variables were forwarded to multivariable binary logistic regression analysis. Variables with a VIF score of ≥ 5 to 10 and tolerance below 0.1 were excluded from the final model. A univariate binary logistic regression analysis was performed to examine the factors associated with Depression and Anxiety in IBD patients. Variables with a *p* value of 0.25 or less in the bivariate analysis were entered into the multivariable binary logistic model. A multiple binary logistic regression model was performed to assess the independent association between factors and depression and anxiety symptoms in IBD patients. The logistic regression results reported as adjusted odds ratios (OR) with 95% confidence intervals and *p* value < 0.05 were considered statistically significant.

## 3 Results

**FIGURE 1:**
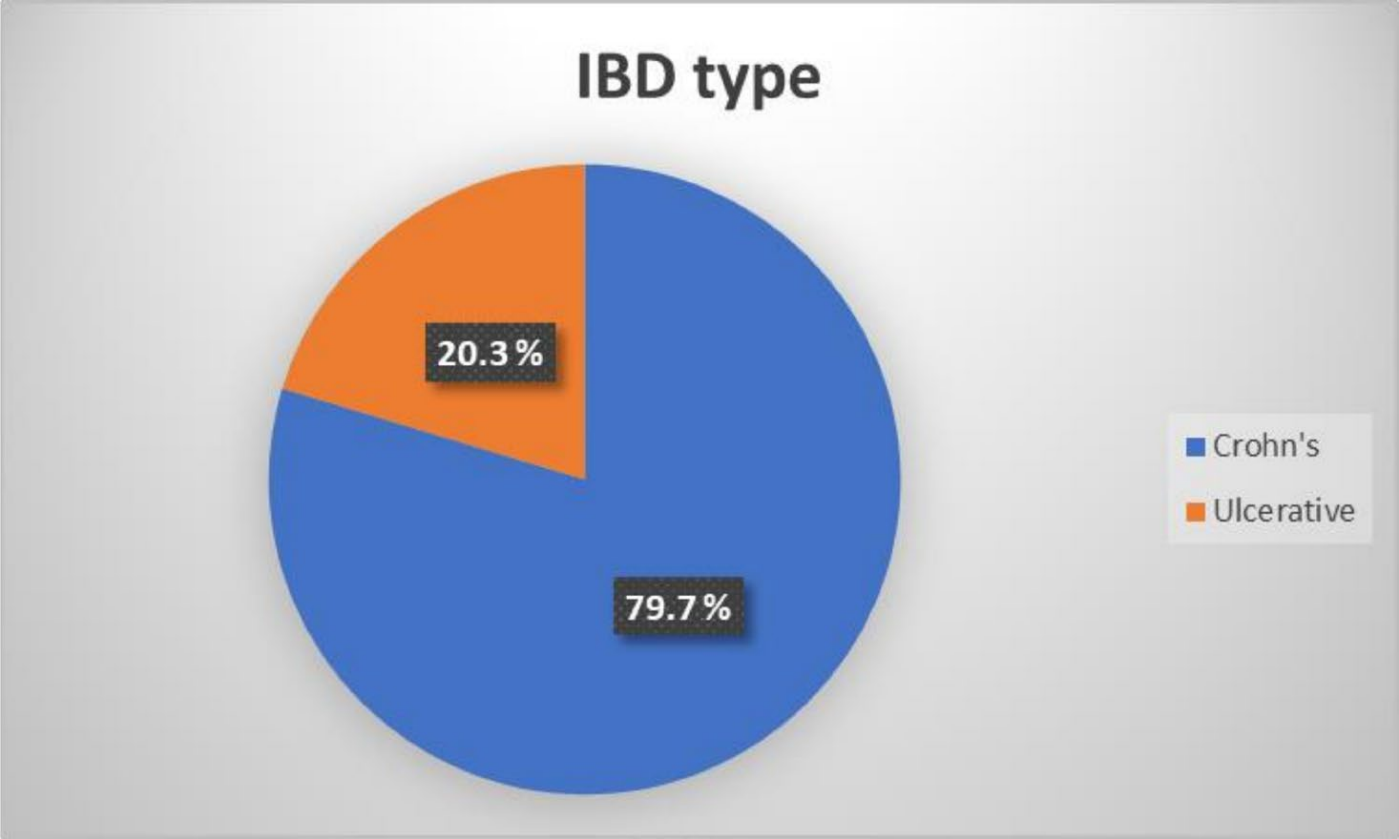
Types of inflammatory bowel diseases.

An analysis of 118 patients revealed a median age of 33 years with an interquartile range of 12.5 years. Among the participants, 67.8% were female, and 53.4% were employed. The majority of patients (79.7%) were diagnosed with Crohn's disease, and 92.4% exhibited mild disease activity. Approximately 35.6% of the patients had undergone previous surgery. In terms of mental health, the prevalence of symptoms of depression and anxiety was found to be 7.6% and 5.1%, respectively. These findings provide valuable insights into the demographics, disease characteristics, and mental health status of the patient population under study (Table 1 and Figure 1).

**TABLE 1:** Sociodemographic characteristics and clinical profile of IBD patients.

Variable	Response	Frequency	Percentage
Age	Median ± IQR	33 ± 12.5 years	
Sex	Female	80	67.8
	Male	38	32.2
Education level	High school or below	60	50.8
	Undergraduate or postgraduate	58	49.2
Marital status	Divorced/widowed/single	68	57.6
	Married	50	42.4
Job	Employed	63	53.4
	Unemployed	55	46.6
Type of IBD	Crohn's disease	94	79.7
	Ulcerative colitis	24	20.3
Smoking status	Smoker	9	7.6
	Non‐smoker	109	92.4
Disease activity	Mild	87	73.7
	Moderate	28	23.7
	Severe	3	2.5
Previous IBD surgery	No	76	64.4
	Yes	42	35.6
Medication	Biologics	1	0.8
	Steroid and/immunomodulator	117	99.2%
Depression	No	109	92.4
	Yes	9	7.6
GAD	No	112	94.9
	Yes	6	5.1

Abbreviations: GAD: general anxiety disorder, IBD: inflammatory bowel disease, IQR: interquartile range.

**FIGURE 2:**
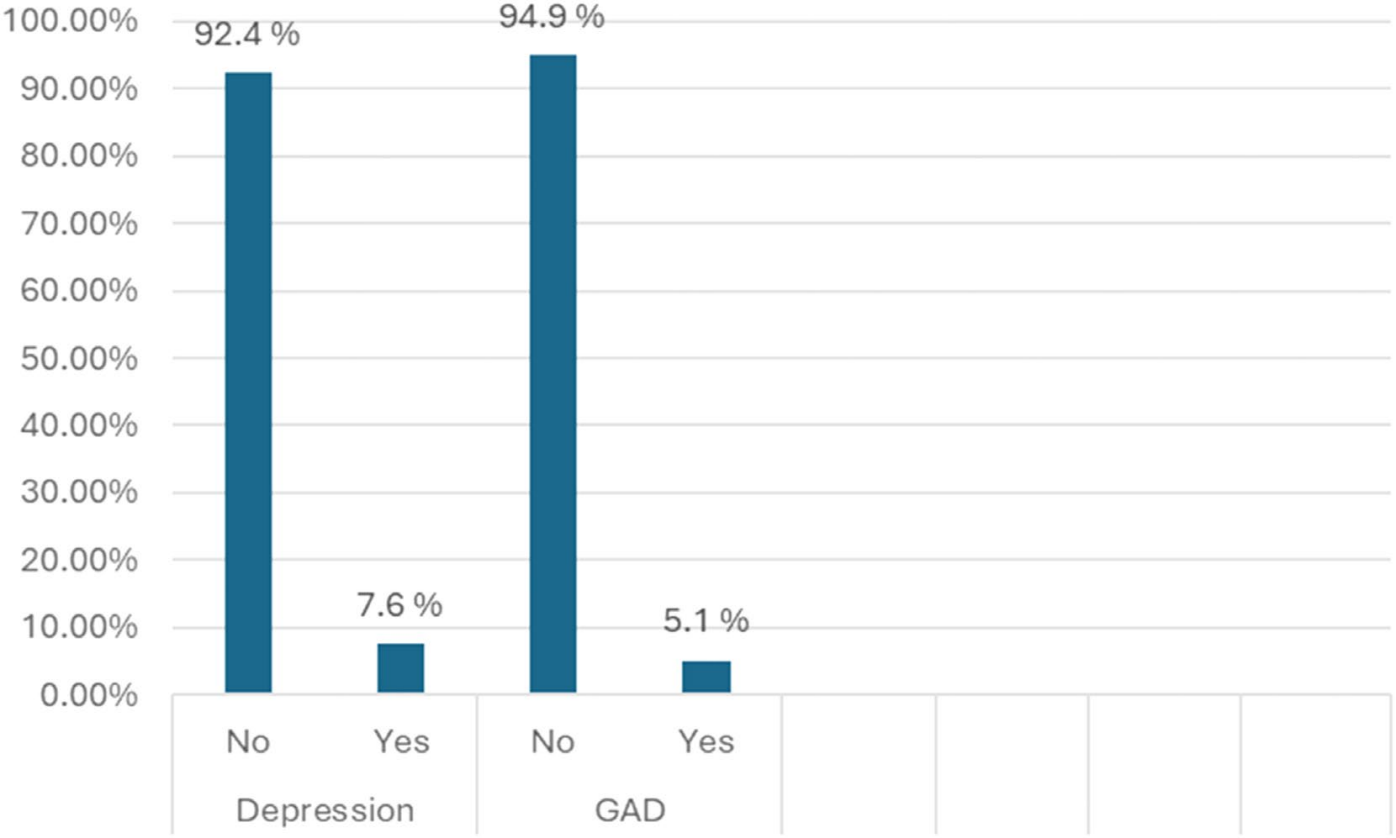
Prevalence of symptoms of depression and anxiety among IBD patients.

The study revealed a 5.1% prevalence of generalized anxiety disorder among individuals with IBD, underscoring the psychological complexities that coexist with the physical manifestations of the disease. Following the univariate analysis, variables identified as relevant, including smoking status and disease activity, were subjected to a multivariate analysis. This comprehensive approach aimed to elucidate the intricate relationship between these factors and anxiety levels in IBD patients. Interestingly, the multivariate logistic regression revealed that smoking did not demonstrate a statistically significant association with anxiety in IBD patients (AOR = 3.4, 95% CI: 0.42, 28.5, *p* = 0.25), emphasizing the need for further investigation into the nuanced interplay between smoking habits and anxiety in IBD patients. The study unearthed compelling insights regarding disease activity levels and their correlation with anxiety in IBD patients. Moderate disease activity (AOR = 9.9, 95% CI: 0.9, 106.2, *p* = 0.058) showed a positive association, while severe disease activity (AOR = 45.3, 95% CI: 2.0, 1018.0, *p* = 0.016) emerged as a statistically significant associated factor with heightened anxiety levels, underlining the clinical importance of disease management strategies aimed at alleviating these symptoms (Table 2 and Figure 2).

**TABLE 2:** factors associated with significant symptoms of depression among IBD patients.

Item	Variables	Depression		COR (95% CI)	*p*	AOR (95% CI)	*p*
		Yes	No				
Smoking status	Smoker	4	5	16.64 (3.39, 81.75)	0.001	1.6 (0.15, 17.22)	0.053
	Non‐smoker	5	104	1		1	
Gender	Female	5	75	1			
	Male	4	34	1.8 (0.45, 6.9)	0.41		
Marital status	Divorced/widowed/single	5	63	1			
	Married	4	46	0.913 (0.22, 3.358)	0.896		
Educational level	High school or below	3	57	0.456 (0.11, 1.82)	0.284		
	Undergraduate or postgraduate	6	52	1			
Job	Employed	4	59	0.578 (0.173, 2.66)	0.578		
	Unemployed	5	50	1			
Type of inflammatory bowel disease	Crohn's disease	6	88	1			
	Ulcerative colitis	3	21	2.10 (0.48, 9.07)	0.323		
Onset of the disease	< 4	7	55	3.44 (0.68, 17.29)	0.134	1.6 (0.49, 17.2)	0.07
	≥ 4	2	54	1			
Disease activity	Mild	1	86	1		1	
	Moderate	7	21	28.6 (3.3, 245.8)	0.002	16.1 (1.7, 156.7)	0.015*
	Severe	1	2	43.0 (1.9, 960.4)	0.018	49.8 (2.1, 1144.02)	0.014*
Previous IBD‐related surgery	No	7	69	2.03 (0.41, 10.24)	0.392		
	Yes	2	40	1			

Abbreviations: AOR: adjusted odds ratio, CI: confidence interval, COR: crude odds ratio, IBD: inflammatory bowel disease.

* Statistically significant.

**FIGURE 3:**
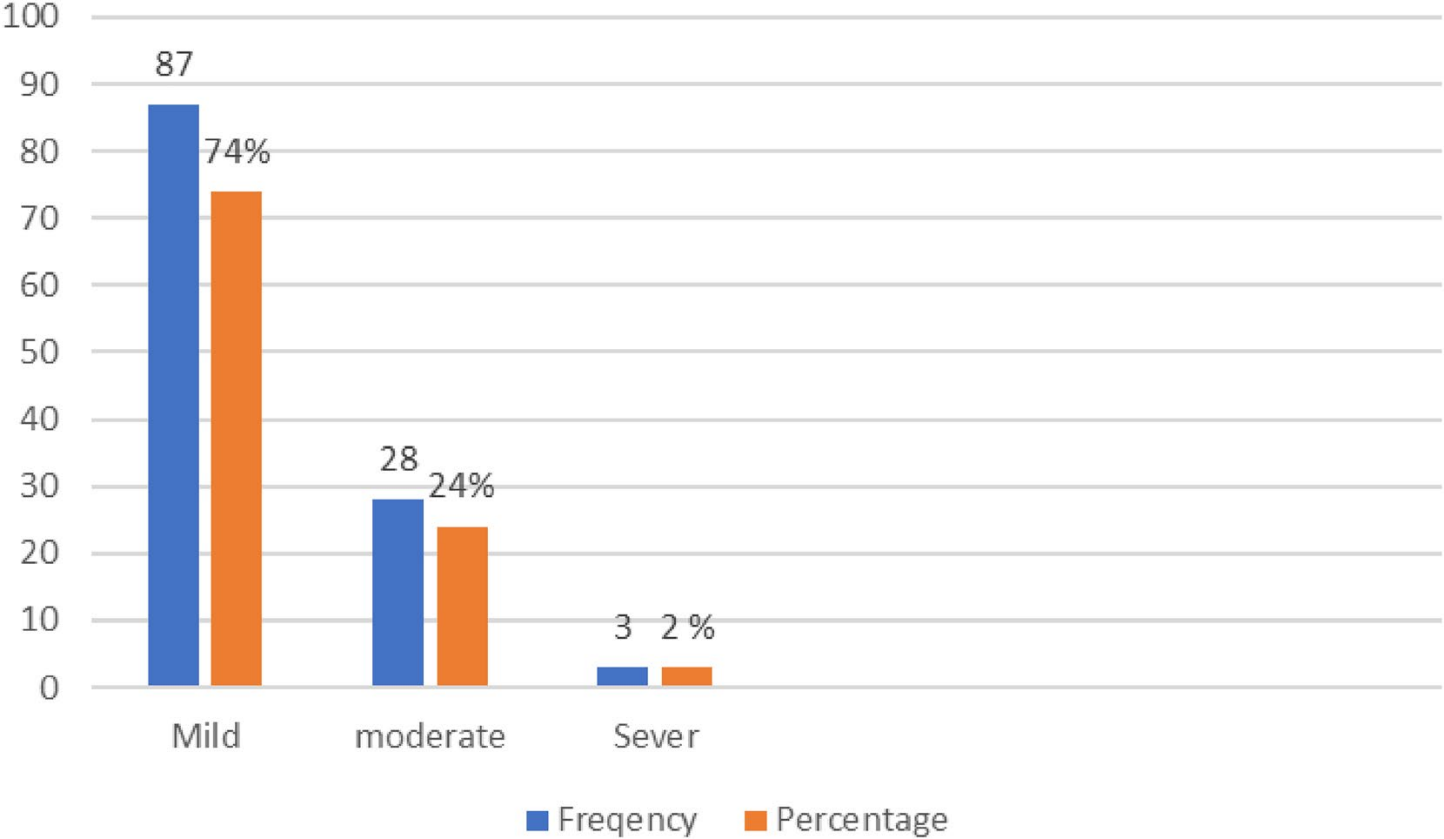
Inflammatory bowel disease activity.

The study also revealed that 7.6% of the patients had significant symptoms of depression. To identify the factors associated with depression in these patients, both univariate and multivariate binary logistic regression analyses were performed. This analysis aimed to determine the various variables that may contribute to the prevalence of significant depressive symptoms in this particular patient population. During the univariate analysis, several factors showed a positive association with increased depression in IBD patients. These factors included smoking status (COR = 16.64 (3.39, 81.75), *p* = 0.001), being male (COR = 1.8 (0.45, 6.9), *p* = 0.41), having ulcerative colitis (COR = 2.10 (0.48, 9.07) *p* = 0.323), having a disease duration of less than 4 years (COR = 3.44 (0.68, 17.29), *p* = 0.132), and having severe disease activity (COR = 43.0 (1.9, 960.4), *p* = 0.018). After adjusting for multiple variables in the multivariate analysis, it was found that moderate disease activity (AOR = 16.1 (1.7, 156.7), *p* = 0.015) and severe disease activity (AOR = 49.8 (2.1, 1144.02), *p* = 0.014) had a statistically significant association with significant depressive symptoms in this population. However, smokers (AOR = 1.6 (0.15, 17.22), *p* = 0.053) and those with a disease duration of less than 4 years (AOR = 1.6 (0.49, 17.2), *p* = 0.07) also showed a positive association with increased depression, though these associations did not reach statistical significance (Table 3 and Figure 3).

**TABLE 3:** Univariate and multivariate binary logistic regression to identify factors associated with significant symptoms of generalized anxiety disorder.

Item	Variables	GAD		COR (95% CI)	*p*	AOR (95% CI)	*p*
		Yes	No				
Smoking status	Smoker	2	7	7.5 (1.17, 48.27)	0.034	3.4 (0.42, 28.5)	0.25
	Non‐smoker	4	105	1		1	
Gender	Female	4	76	0.95 (0.17, 5.41)	0.952		
	Male	2	36	1			
Marital status	Divorced/widowed/single	4	64	1.5 (0.26, 8.53)	0.648		
	Married	2	48	1			
Educational level	High school or below	3	57	0.965 (0.19, 4.99)	0.966		
	Undergraduate or postgraduate	3	55	1			
Job	Employed	2	61	0.42 (0.07, 2.38)	0.325		
	Unemployed	4	51	1			
Type of inflammatory bowel disease	Crohn's disease	5	89	1.29 (0.44, 11.06)	0.819		
	Ulcerative colitis	1	23	1			
The year since the onset of the disease	< 4	5	74	2.57 (0.29, 22.76)	0.397		
	≥ 4	1	38	1			
Disease activity	Mild	1	86	1			
	Moderate	4	24	14.333 (1.53, 134.06)	0.020	9.9 (0.9, 106.2)	0.058
	Severe	1	2	43.0 (1.93, 960.2)	0.018	45.3 (2.0, 1018.0)	0.016*
Previous IBD‐related surgery	No	5	71	2.887 (0.326, 25.57)	0.341		
	Yes	1	41	1			

Abbreviations: AOR: adjusted odds ratio, CI: confidence interval, COR: crude odds ratio, GAD: generalized anxiety disorder, IBD: inflammatory bowel disease.

* Statistically significant.

## 4 Discussion

This study offers valuable insights into the prevalence of mental health symptoms among individuals with IBD within high‐volume medical treatment sites. The research revealed symptoms of depression and anxiety to be 7.6% and 5.1%, respectively. Interestingly, these prevalence rates were notably lower when contrasted with findings from other observational studies and meta‐analyses. The reported prevalence of anxiety (5.1%) and depression (7.6%) in our research is notably lower than figures found in global meta‐analyses, which often indicate much higher rates of psychiatric comorbidities in patients with IBD. Massironi et al. highlighted a significantly higher prevalence, with pooled estimates of depression (OR 1.42) and anxiety (OR 1.3) [10]. Several factors may explain this discrepancy. Cultural attitudes toward mental health in Ethiopia may lead to underreporting of psychiatric symptoms, as mental health issues can carry stigma, resulting in patients being less likely to disclose their experiences. Additionally, differences in healthcare access and the availability of mental health resources may influence diagnosis and treatment rates. The characteristics of our sample population, including socioeconomic status and disease severity, may also differ from those in studies conducted in higher‐income countries, which could further impact the observed prevalence rates. These factors underscore the need for culturally sensitive screening and assessment methods to better understand and address the mental health needs of IBD patients in diverse settings.

A systematic review and meta‐analysis conducted in 2021 reported that approximately 35% of patients with IBD experience anxiety symptoms, while around 22% report depressive symptoms. This underscores the high burden of mental health issues in this population, which is often overlooked in clinical settings [3]. Research by Fakhfouri et al. has shown that the severity of IBD is closely linked to the prevalence of psychiatric disorders. Patients with active disease are more likely to experience anxiety and depression, suggesting that effective management of IBD may also alleviate mental health symptoms [3].

A meta‐analysis of five observational and population‐based studies, including 767 patients, found that the pooled mean rate of depressive symptoms across all IBD samples reporting percentages was approximately 21%. In a similar study, the mean percentage of anxiety symptoms in all IBD cases was 19% [9, 13]. In another meta‐analysis of 171 studies totaling 158,371 participants, it was found that approximately 35% of IBD patients experienced symptoms of anxiety, while 22% reported symptoms of depression [6, 9]. Another extensive global evidence, in a meta‐analysis of 58 observational studies, was discovered that the pooled prevalence of anxiety symptoms was roughly 32%, and in a meta‐analysis of 75 observational studies, the combined prevalence of depression symptoms was 25% [8, 9]. Various factors have been linked to heightened psychiatric disorders in individuals with IBD [14]. In our study, we found that disease activity, smoking, unemployment, short duration after diagnosis, ulcerative IBD, and being unmarried are positively associated with depression and anxiety in IBD patients. A recent systematic review with meta‐analysis by Massironi et al. also showed that active disease stages are associated with higher rates of depression and anxiety [10]. This underscores the importance of disease management in addressing mental health, suggesting integrated care approaches that prioritize mental health alongside physical health.

A study involving 1663 IBD patients revealed that severe disease, flare‐ups, noncompliance with treatment, being disabled or unemployed, and experiencing socioeconomic deprivation were all correlated with increased rates of depression and anxiety [14]. In research conducted in Korea, 369 patients suffering from IBD were examined. The study revealed that factors like marital status, the use of anti‐tumor necrosis factor agents, age, body mass index, disease activity, alcohol consumption, and employment status were linked to increased levels of depression and anxiety in individuals with IBD [15]. Moreover, extensive longitudinal research has consistently indicated that individuals experiencing symptoms of anxiety and depression disorders tend to have more frequent flares of IBD and experience worse disease activity [16]. In summary, factors such as the emergence of a new disease, disease activity levels, medication side effects, stressful life events, hospitalization, and lower socioeconomic status can significantly impact the mental well‐being of individuals with one or both types of IBD [17].

A systematic review in 2021 assessed cognitive impairment in adults with IBD, revealing significant associations between cognitive deficits and psychiatric disorders. This relationship complicates disease management and emphasizes the importance of addressing both cognitive and emotional health in IBD patients [3]. Recent longitudinal studies have indicated that mental health issues often develop or worsen after an IBD diagnosis. This finding suggests that healthcare providers should implement continuous mental health monitoring and support as part of routine care for IBD patients [3]. Given these findings, healthcare providers must adopt a holistic approach to managing IBD that includes regular mental health screenings. Utilizing validated tools such as the Generalized Anxiety Disorder Scale (GAD‐7) and the Patient Health Questionnaire (PHQ‐9) can help identify patients at risk for psychiatric comorbidities early in their treatment. Furthermore, integrating mental health professionals into the care team can facilitate comprehensive treatment plans that address both physical and psychological aspects of IBD. In summary, the evolving understanding of the relationship between IBD and psychiatric comorbidities necessitates an updated approach to patient care. By prioritizing mental health alongside physical health, healthcare providers can significantly improve the quality of life and treatment outcomes for individuals living with IBD.

The observed associations between disease activity and symptoms of anxiety and depression in patients with IBD can be understood through various biological and psychological pathways.

Chronic inflammation, a hallmark of IBD, is known to influence mental health through the release of pro‐inflammatory cytokines, such as interleukin‐6 and tumor necrosis factor‐alpha. These cytokines can affect neurotransmitter systems, including serotonin and dopamine, which are critical for regulating mood and anxiety [18]. Additionally, the gut‐brain axis plays a crucial role in mental health, where alterations in gut microbiota due to inflammation can impact brain function and behavior, potentially leading to increased anxiety and depressive symptoms [3]. The release of these inflammatory mediators can lead to neuroinflammation, which has been linked to the development of depressive symptoms [18].

From a psychological standpoint, the experience of living with a chronic illness like IBD can significantly affect an individual's quality of life. Fluctuations in disease activity often lead to uncertainty about the future, social withdrawal, and feelings of helplessness, which can exacerbate anxiety and depressive symptoms [19]. Moreover, the burden of managing a chronic illness may hinder effective coping strategies, leading to increased stress and mental health challenges [19]. Studies have shown that depression can independently predict poorer IBD outcomes, suggesting a bidirectional relationship where mental health issues can worsen disease activity [3]. Understanding these interconnected pathways is essential for developing comprehensive treatment approaches that address both the physical and psychological aspects of IBD. By recognizing the role of disease activity in exacerbating mental health issues, healthcare providers can implement targeted interventions, such as psychological support and inflammatory control strategies, to improve overall patient outcomes [3, 18].

The findings of this study underscore the importance of integrating mental health management into the clinical care of patients with IBD. Given the observed associations between disease activity and symptoms of anxiety and depression, healthcare providers should prioritize mental health screenings as part of routine assessments for IBD patients.

### 4.1 Integrated Care Approach

An integrated care model that addresses both physical and mental health is crucial for improving patient outcomes. Clinicians should be trained to recognize the signs of anxiety and depression and utilize validated screening tools, such as the Generalized Anxiety Disorder Scale (GAD‐7) and the Patient Health Questionnaire (PHQ‐9), to identify patients at risk. Early identification of psychological distress can facilitate timely interventions, such as counseling or referral to mental health professionals.

### 4.2 Patient Education and Support

Educating patients about the potential psychological impacts of IBD is essential. Providing information on the links between disease activity and mental health can empower patients to discuss their symptoms openly. Support groups and educational workshops can also be valuable resources, offering patients a platform to share experiences and coping strategies.

### 4.3 Holistic Treatment Plans

Management strategies should encompass not only pharmacological treatments for IBD but also therapeutic options for mental health. Cognitive‐behavioral therapy (CBT) and mindfulness‐based interventions have shown promise in alleviating anxiety and depression symptoms in chronic illness populations. Collaborating with mental health specialists can help develop comprehensive treatment plans that address the multifaceted needs of IBD patients.

### 4.4 Continuous Monitoring

Ongoing monitoring of mental health status should be part of follow‐up care. Regular assessments can help track changes in anxiety and depression symptoms about disease activity, ensuring that appropriate interventions are implemented when needed. This proactive approach can lead to better management of both IBD and mental health conditions, ultimately enhancing the quality of life for patients.

### 4.5 Policy Implications

At a broader level, the findings highlight the need for healthcare policies that promote mental health services within IBD care frameworks. Policymakers should advocate for increased funding and resources dedicated to mental health support for chronic illness patients, recognizing the significant impact that mental health has on overall health outcomes.

In summary, the integration of mental health management into the care of IBD patients is vital. By adopting a holistic approach that includes regular screening, patient education, and interdisciplinary collaboration, healthcare providers can significantly improve the quality of life and treatment outcomes for individuals living with IBD.

## 5 Limitations of the Study

This study has several limitations that should be acknowledged, which may affect the interpretation and generalizability of the findings.

This study is limited to two medical centers in Ethiopia, which may affect the generalizability of the findings to other populations, particularly those in different socio‐economic and cultural contexts. The unique healthcare environment and specific characteristics of the patient population at these centers may not fully represent the broader IBD population in Ethiopia or the surrounding region. Consequently, the observed prevalence rates of anxiety and depression may differ in other settings, where variations in healthcare access, cultural perceptions of mental health, and socio‐economic conditions could influence outcomes. Additionally, the study's design might introduce selection bias, as patients attending these centers may not reflect the experiences of those who do not seek care. This could lead to an underrepresentation of more severe cases or individuals with significant psychiatric comorbidities, resulting in an artificially low prevalence of anxiety and depression.

Future research should aim to include a more diverse array of medical centers and regions to enhance the generalizability of findings and better understand the mental health implications of IBD across different contexts.

The sample size of 118 patients in this study is relatively small compared to other large‐scale studies in the field, which may limit the robustness and generalizability of our findings. The observed wide confidence intervals for some statistical results (e.g., AOR = 49.8, 95% CI: 2.1, 1144.02) suggest potential instability in these estimates, which could be attributed to the small sample size and low event rates for anxiety and depressive symptoms. This limitation should be taken into consideration when interpreting the results, as it may affect the statistical power and the precision of our conclusions regarding the associations between disease activity and mental health outcomes in IBD patients. Future studies with larger sample sizes are warranted to validate these findings and provide more reliable estimates.

The cross‐sectional nature of the study means that causality cannot be established. While associations between disease activity and mental health symptoms were observed, it is unclear whether high disease activity leads to increased anxiety and depression or if pre‐existing mental health issues exacerbate disease symptoms. Longitudinal studies are needed to clarify these relationships.

The reliance on self‐reported questionnaires, such as the GAD‐7 and PHQ‐9, may introduce response bias. Participants might underreport symptoms due to the stigma associated with mental health or misinterpret the questions. This limitation highlights the need for clinical assessments to complement self‐reported measures.

By acknowledging these limitations, we aim to provide a clearer understanding of the study's findings and their implications for the management of mental health in patients with IBD. Future research should address these limitations by incorporating larger, more diverse samples and longitudinal designs to enhance the validity and applicability of the results.

## 6 Conclusion

This study highlights the significant prevalence of anxiety and depression among patients with IBD in Ethiopia, emphasizing the need for integrated mental health care in this population. Our findings reveal a concerning association between disease activity and psychiatric comorbidities, underscoring the importance of routine mental health screenings in clinical practice. This study contributes to the existing literature by focusing on a unique patient population in a low‐resource setting, a context that has been underrepresented in prior research. By examining mental health outcomes in IBD patients in Ethiopia, we provide valuable insights into how cultural perceptions of mental health may influence the experiences of these individuals. Additionally, our methodology, which incorporates validated screening tools adapted for the local context, enhances the reliability of our findings. This regional focus not only highlights the need for tailored mental health interventions but also underscores the importance of understanding the interplay between sociocultural factors and mental health in chronic illness management.

Our findings call for further research in diverse populations to develop comprehensive care strategies that address both physical and psychological health in IBD patients globally.

## Ethics Statement

The study was conducted by the Declaration of Helsinki and approved by the Institutional Review Board of Addis Ababa University, College of Medicine and Health Sciences.

## Consent

Informed consent was obtained from all subjects involved in the study.

## Conflicts of Interest

The authors declare no conflicts of interest.

## Data Availability

The data that support the findings of this study are available on request from the corresponding author. The data are not publicly available due to privacy or ethical restrictions.
